# Supplementary material for: Persistence against benzalkonium chloride promotes rapid evolution of tolerance during periodic disinfection
Source: Nat Commun. 2021 Nov 23;12:6792. doi: 10.1038/s41467-021-27019-8 (PMC8611074; doi:10.1038/s41467-021-27019-8)
Supplement: Supplementary file 1 — Supplementary Information [file 41467_2021_27019_MOESM1_ESM.pdf]

Supplementary Information

Persistence against benzalkonium chloride promotes rapid evolution of tolerance during periodic disinfection

Niclas Nordholt[a#], Orestis Kanaris[a], Selina B.I. Schmidt[a], Frank Schreiber[a#]

[a]Division of Biodeterioration and Reference Organisms (4.1), Department of Materials and the Environment, Federal Institute for Materials Research and Testing (BAM), Berlin, Germany

[#]Address correspondence to Niclas Nordholt and Frank Schreiber, niclas.nordholt@bam.de and frank.schreiber@bam.de; Tel.: +49-30-8104-1414

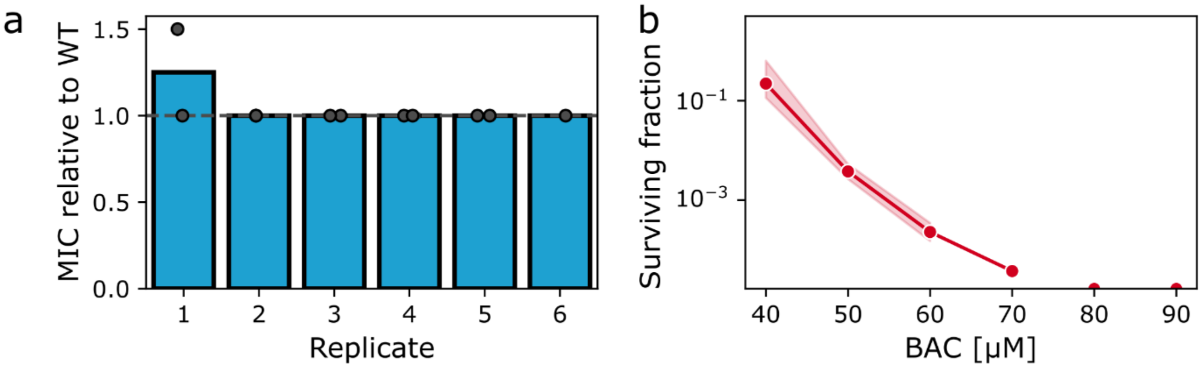

Figure S1: a) BAC persisters are not resistant mutants. *E. coli* was exposed to 60 µM BAC. Per biological replicate, two colonies of surviving cells after 15 minutes of BAC treatment were picked and the MIC of BAC was determined. **b)** Concentration dependence of persisters after 20 minutes of BAC treatment (Spearman correlation coefficient -0.958, $p = 2\times10^{-7}$, n = 3 biological replicates. Data points show the geometric mean ± 95% confidence intervals. Significance of correlation: two-sided test with t-distribution of the test statistic). This figure accompanies figure 1. Source data are provided as a Source Data file.

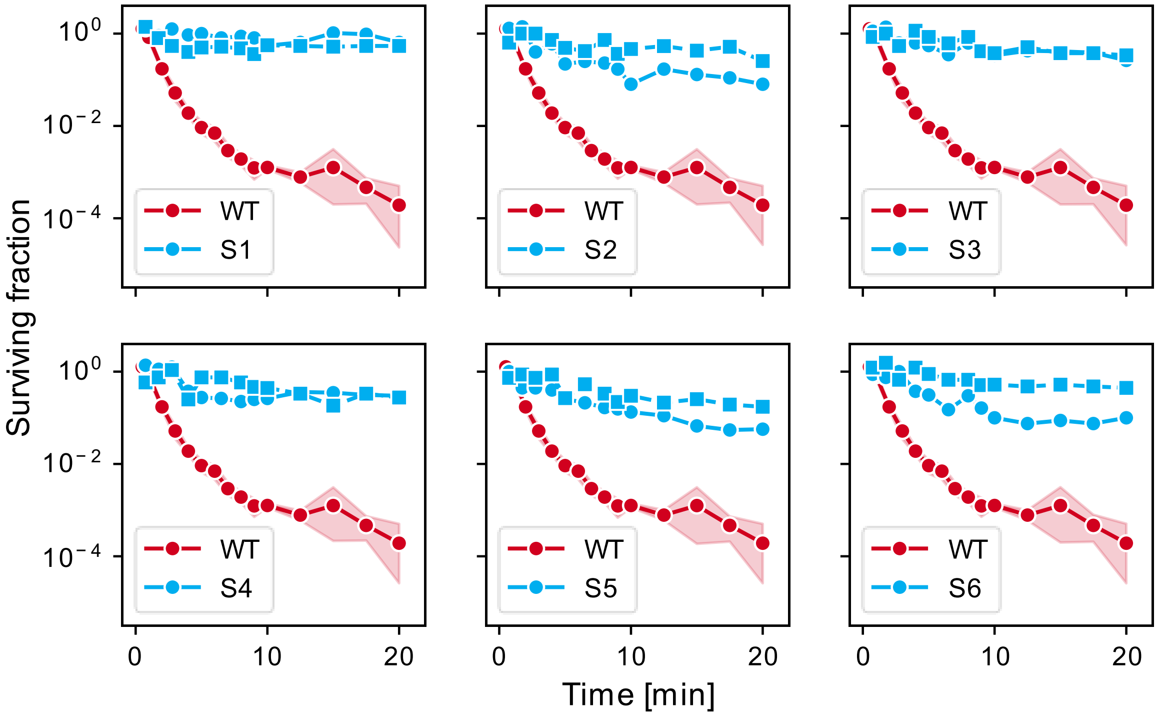

Figure S2: Time-kill curves of evolved clones. Blue symbols represent individual replicates of the surviving fraction (n=2 biological replicates). For the wild-type (WT), the symbol represents the geometric mean and the shaded area represents the 95% confidence interval (n=6 biological replicates). The red curve is the same as in figure 1 A in the main text. This figure accompanies figure 2. Source data are provided as a Source Data file.

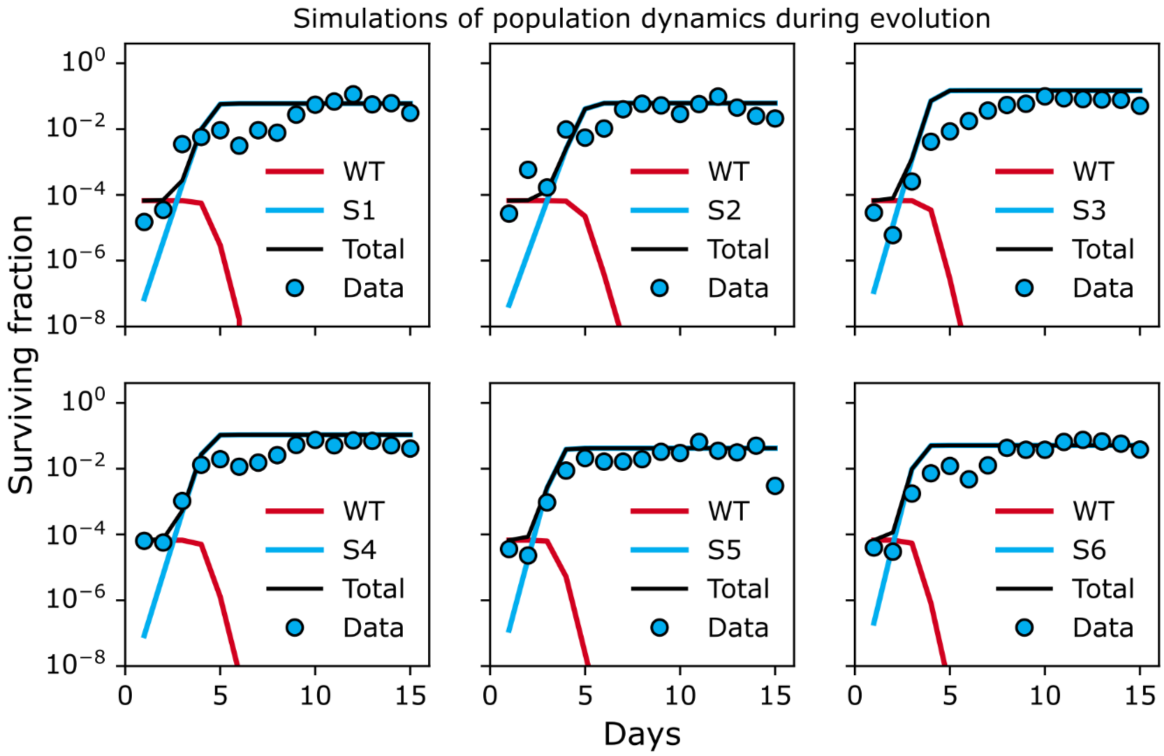

Figure S3: Simulations of the population dynamics during evolution for all evolved clones. Simulations of survival fraction during the serial passage experiment with alternating growth and kill cycles (lines) quantitatively reproduce the experimental data (circles). The parameters for growth rate and survival fraction are the same as in figure 2 C. Yield was inferred from cfu to OD_{600} conversion factors of the individual strains. Initial number of ancestor and mutant cells: 10^5 and 1 respectively. This figure accompanies figure 2. Source data are provided as a Source Data file.

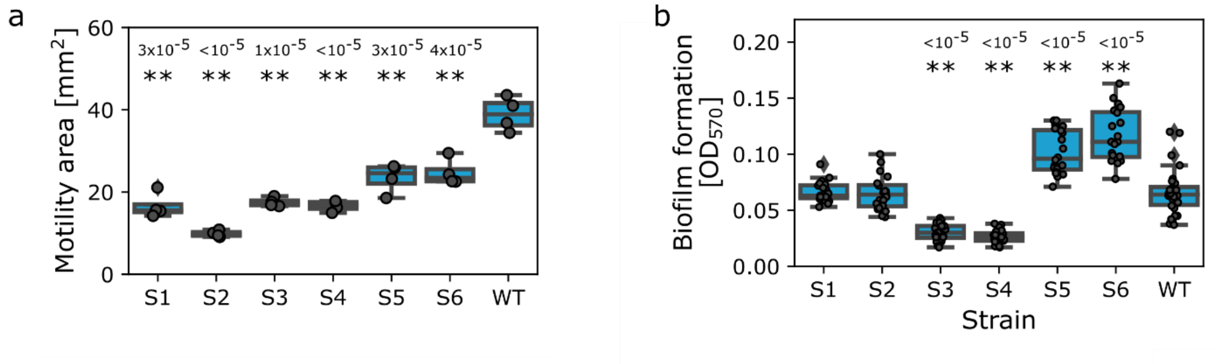

Figure S4: Motility and biofilm formation are affected int the evolved clones. a) Motility is decreased in all evolved clones. Boxes denote the interquartile range (IQR) between the first and third quartile, with the line showing the median. Whiskers indicate the minima and maxima of the data or have a length of 1.5 IQR if data fall outside the 1.5 IQR. n = 4 biological replicates. Significance of motility area against ancestor: *, $p < 0.05$; **, $p < 0.01$ (one-tailed t-test of motility area in mm^2). **b)** Evolved BAC tolerance affects biofilm formation. Boxes denote the IQR, with the line showing the median. Whiskers indicate the minima and maxima of the data or have a length of 1.5 IQR if data fall outside the 1.5 IQR. n = 20 biological replicates. Significance of biofilm formation against the ancestor is indicated by asterisks: *, $p < 0.05$; **, $p < 0.01$ (two-tailed t-test of optical density at 570 nm). Exact p-values are indicated above the asterisks. Source data are provided as a Source Data file.

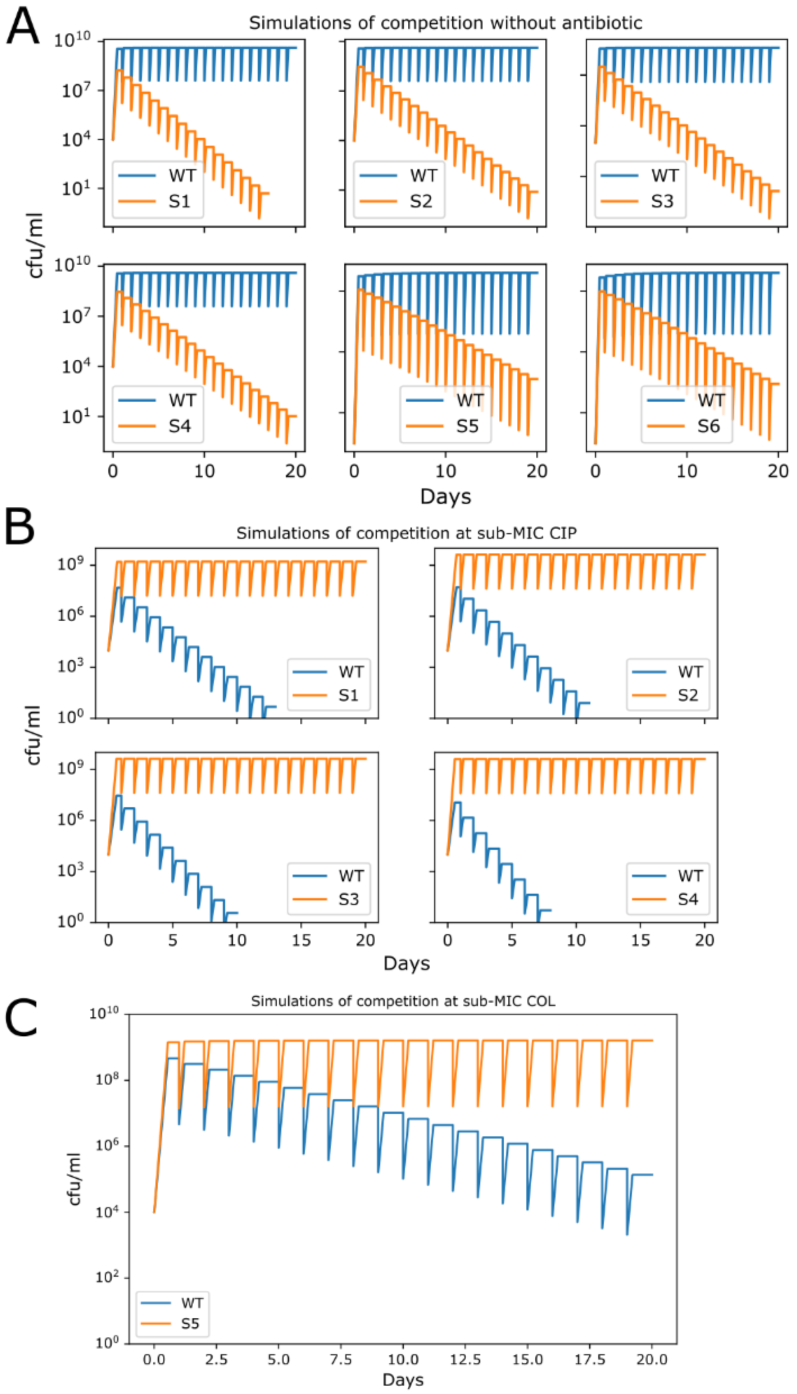

Figure S5: Simulations of competition experiments in the absence and presence of sub-inhibitory antibiotic concentrations. A) When competing in the absence of antibiotics, the wild-type wins over the evolved clones due to the costs of tolerance. **B)** In the presence of ciprofloxacin, four of the evolved clones outcompete the ancestor within 7 – 12 growth cycles. **C)** S5 outcompetes the wild-type in sub-inhibitory levels of colistin. Simulations are shown for strains with a growth-rate significantly different from the wild-type (see Figure 2C and Figure 5B in the main text). This figure accompanies figure 5 in the main text.

Table S1: Mutations in all evolved clones generated in this study.

Strain	Genomic position	Mutation	Amino acid substitution	Gene(s)	Annotation
**S1c1** S1c2	1,094,727	Δ26,518 bp	n.a.	*ymdE, ycdU, serX, ghrA, ycdX, ycdY, ycdZ, csgG, csgF, csgE, csgD, csgB, csgA, csgC, ymdA, ymdB, clsC, opgC, opgG, opgH, yceK, msyB, mdtG, lpxL, yceA, yceI, yceJ, yceO, solA, bssS, dinI, [pyrC]*	IS3-mediated See Table S2 for details
	1,939,467	G→A	intergenic (-50/+70)	*lpxM* ← / ← *mepM*	myristoyl-acyl carrier protein-dependent acyltransferase/peptidoglycan DD-endopeptidase MepM
**S2c1** S2c2	1,115,520	G→T	A96E	*lpxL* ←	lauroyl acyltransferase
	1,971,413	Δ6,314 bp	n.a.	*[tar], cheW, cheA, motB, motA, flhC, flhD*	IS1-mediated See Table S2 for details
**S3c1**	1,289,634	T→C	C57R	*rssB* →	regulator of RpoS
	1,938,693	G→T	A242E	*lpxM* ←	myristoyl-acyl carrier protein-dependent acyltransferase
S3c2	1,938,874	C→T	G182S	*lpxM* ←	myristoyl-acyl carrier protein-dependent acyltransferase
	1,967,842	*Δ9,885 bp*	*n.a.*	*[cheR], tap, tar, cheW, cheA, motB, motA, flhC, flhD*	IS1-mediated See Table S2 for details
S4c1	1,290,339	C→T	Q292*	*rssB* →	regulator of RpoS
	1,938,979	C→A	A147S	*lpxM* ←	myristoyl-acyl carrier protein-dependent acyltransferase
**S4c2**	1,290,339	C→T	Q292*	*rssB* →	regulator of RpoS
	1,938,975	A→C	M148R	*lpxM* ←	myristoyl-acyl carrier protein-dependent acyltransferase
**S5c1** S5c2	1,108,517	+9 bp	intergenic (-352/-34)	*opgC* ← / → *opgG*	protein required for succinyl modification of osmoregulated periplasmic glucans/osmoregulated periplasmic glucans (OPGs) biosynthesis protein G

	1,938,799	A→T	L207I	*lpxM* ←	myristoyl-acyl carrier protein-dependent acyltransferase
S6c1	1,111,908	Δ2 bp	coding (1822-1823/ 2544 nt)	*opgH* →	osmoregulated periplasmic glucans (OPGs) biosynthesis protein H
	1,938,526	A→C	W298G	*lpxM* ←	myristoyl-acyl carrier protein-dependent acyltransferase
	4,123,417	*Δ13 bp*	*intergenic (-78/+2)*	*ftsN ← / ← cytR*	*cell division protein FtsN/DNA-binding transcriptional repressor CytR*
**S6c2**	1,111,908	Δ2 bp	coding (1822-1823/ 2544 nt)	*opgH* →	osmoregulated periplasmic glucans (OPGs) biosynthesis protein H
	1,938,526	A→C	W298G	*lpxM* ←	myristoyl-acyl carrier protein-dependent acyltransferase

n.a., not applicable; bold typeface indicates clones selected for further analyses in the main text; italic font indicates rare mutations that were not present in the population sequencing approach

Table S2: Genes affected by large IS-mediated deletions.

Clone	Gene	Annotation
S1	*ymdE*	Uncharacterized protein YmdE
	ycdU	Uncharacterized protein YcdU
	serX	tRNA-Ser
	ghrA	glyoxylate/hydroxypyruvate reductase A
	ycdX	zinc-binding phosphatase
	ycdY	chaperone protein YcdY
	ycdZ	putative inner membrane protein
	csgG	curli secretion channel
	csgF	curli assembly component
	csgE	curli transport specificity factor
	csgD	DNA-binding transcriptional dual regulator CsgD
	csgB	curlin minor subunit
	csgA	curlin major subunit
	csgC	inhibitor of CsgA amyloid formation
	ymdA	uncharacterized protein YmdA
	ymdB	2'-O-acetyl-ADP-ribose deacetylase regulator of RNase III activity
	clsC	cardiolipin synthase C
	opgC	protein required for succinyl modification of osmoregulated periplasmic glucans
	opgG	osmoregulated periplasmic glucans (OPGs) biosynthesis protein G
	opgH	osmoregulated periplasmic glucans (OPGs) biosynthesis protein H
	yceK	DUF1375 domain-containing lipoprotein YceK
	msyB	acidic protein that suppresses heat sensitivity of a secY mutant
	mdtG	efflux pump MdtG
	lpxL	lauroyl acyltransferase
	yceA	UPF0176 protein YceA
	yceI	Uncharacterized protein YceI
	yceJ	putative cytochrome b561
	yceO	DUF2770 domain-containing protein YceO
	solA	N-methyl-L-tryptophan oxidase
	bssS	regulator of biofilm formation
	dinI	DNA damage-inducible protein I
	pyrC	dihydroorotase
S2	*tar*	methyl-accepting chemotaxis protein Tar
	cheW	chemotaxis protein CheW
	cheA	chemotaxis protein CheA
	motB	motility protein B
	motA	motility protein A
	flhC	DNA-binding transcriptional dual regulator FlhC
	flhD	DNA-binding transcriptional dual regulator FlhD
S3c2	*cheR*	chemotaxis protein methyltransferase
	tap	methyl-accepting chemotaxis protein Tap
	tar	methyl-accepting chemotaxis protein Tar
	cheW	chemotaxis protein CheW
	cheA	chemotaxis protein CheA
	motB	motility protein B
	motA	motility protein A
	flhC	DNA-binding transcriptional dual regulator FlhC
	flhD	DNA-binding transcriptional dual regulator FlhD

Table S3: Bacterial strains used in this study.

Designation in main text	Designation in original publication	Description	Genotype	Source, Citation
MG1655 (wild-type)	K-12 MG1655	Wild type	Δ776 bp insB9–[crl], +8 bp bamD → / → raiA, +GC gltP → / ← yjcO compared to NC_000913.3	R. Mutzel lab [1]
Δ*tolC*	TB283	Knock-out of the multi-drug efflux channel *tolC*	MG1655 *attP21::PR-mCherry::frt ΔtolC::FRT*	C. Guet lab [2]
ΔrelAspoT	*ΔrelA ΔspoT*	Double knockout of the (p)ppGpp synthase/hydrolase *relA spoT*	MG 1655 *ΔrelA::frt ΔspoT::frt*	K. Lewis lab [3]
P_{lac}-*marA*	*MarA*-CFP	Multi-antibiotic resistance transcription factor *marA* under control of an IPTG-inducible promoter	MG1655 pBbA5k *marA*-CFP	M. Dunlop lab [4]
Δ*marRAB*	Δ*marRAB*	Knockout of the *marRAB* operon. Contains a low copy plasmid with CFP under the control of the *marA* promoter.	MG1655 *ΔmarRAB* pBbSVk-P_{marA}-CFP	M. Dunlop lab [4]
P_{lac}-*tisB*	pZS*24*tisB*	Persistence-inducing toxin *tisB* under control of an IPTG-inducible promoter	MG1655 pZS*24*tisB*	K. Lewis lab [5]
P_{BAD}-hokB	PBAD/*HisA-hokB*	Persistence-inducing toxin *hokB* under control of an arabinose-inducible promoter	MG1655 PBAD/*HisA-hokB*	J. Michiels lab [6]
Δ*fliC*	TB205	Knock-out of the flagellin gene *fliC*	MG1655 *attP21::PR-mCherry::FRT ΔfliC::FRT*	C. Guet lab [2]
P_{lac}-*lpxM*	JW1844-AM	Lipid A biosynthesis myristoyltransferase *lpxM* under control of an IPTG-inducible promoter	MG1655 pCA24N-Plac/T5_6His-*lpxM*	ASKA collection [7]
P_{lac}-*rpoE*	JW2557-AM	Envelope-stress sigma factor *rpoE* under control of an IPTG-inducible promoter	MG1655 pCA24N-Plac/T5_6His-*rpoE*	ASKA collection [7]
Δ*lpxM*	BW25113 *lpxM::FRT-Kan-FRT*	Knock-out of lipid A biosynthesis myristoyltransferase *lpxM*	BW25113 *lpxM::FRT-Kan-FRT*	Keio collection [8]

Table S3: Bacterial strains used in this study.

Designation in main text	Designation in original publication	Description	Genotype	Source, Citation
BW25113 (wild-type)	BW25113		*rrnB3 ΔlacZ4787 hsdR514 Δ(araBAD)567 Δ(rhaBAD)568 rph-1.*	Keio collection [8]

References

1. Blattner, F. R. *et al.* The complete genome sequence of Escherichia coli K-12. *Science* **277**, 1453–1462 (1997).

2. Bergmiller, T. *et al.* Biased partitioning of the multidrug efflux pump AcrAB-TolC underlies long-lived phenotypic heterogeneity. *Science* **356**, 311–315 (2017).

3. Shan, Y. *et al.* ATP-Dependent persister formation in Escherichia coli. *mBio* **8**, e02267-16 (2017).

4. Meouche, I. E., Siu, Y. & Dunlop, M. J. Stochastic expression of a multiple antibiotic resistance activator confers transient resistance in single cells. *Sci. Rep.* **6**, 1–9 (2016).

5. Dörr, T., Vulić, M. & Lewis, K. Ciprofloxacin causes persister formation by inducing the TisB toxin in Escherichia coli. *PLoS Biol.* **8**, e1000317 (2010).

6. Verstraeten, N. *et al.* Obg and Membrane Depolarization Are Part of a Microbial Bet-Hedging Strategy that Leads to Antibiotic Tolerance. *Mol. Cell* **59**, 9–21 (2015).

7. Kitagawa, M. *et al.* Complete set of ORF clones of Escherichia coli ASKA library (A Complete S et of E. coli K -12 ORF A rchive): Unique Resources for Biological Research. *DNA Res.* **12**, 291–299 (2005).

8. Baba, T. *et al.* Construction of Escherichia coli K-12 in-frame, single-gene knockout mutants: the Keio collection. *Mol. Syst. Biol.* **2**, 2006.0008 (2006).
