# Supplementary material for: Persistence against benzalkonium chloride promotes rapid evolution of tolerance during periodic disinfection
Source: Nat Commun. 2021 Nov 23;12:6792. doi: 10.1038/s41467-021-27019-8 (PMC8611074; doi:10.1038/s41467-021-27019-8)
Supplement: Supplementary file 3 — Reporting Summary [file 41467_2021_27019_MOESM3_ESM.pdf]

nature research

Corresponding author(s): Nordholt, Schreiber

Last updated by author(s): Oct 15, 2021

Reporting Summary

Nature Research wishes to improve the reproducibility of the work that we publish. This form provides structure for consistency and transparency in reporting. For further information on Nature Research policies, see our Editorial Policies and the Editorial Policy Checklist.

Statistics

For all statistical analyses, confirm that the following items are present in the figure legend, table legend, main text, or Methods section.

n/a	Confirmed	
☐	☒	The exact sample size (n) for each experimental group/condition, given as a discrete number and unit of measurement
☐	☒	A statement on whether measurements were taken from distinct samples or whether the same sample was measured repeatedly
☐	☒	The statistical test(s) used AND whether they are one- or two-sided<br>*Only common tests should be described solely by name; describe more complex techniques in the Methods section.*
☒	☐	A description of all covariates tested
☐	☒	A description of any assumptions or corrections, such as tests of normality and adjustment for multiple comparisons
☐	☒	A full description of the statistical parameters including central tendency (e.g. means) or other basic estimates (e.g. regression coefficient) AND variation (e.g. standard deviation) or associated estimates of uncertainty (e.g. confidence intervals)
☐	☒	For null hypothesis testing, the test statistic (e.g. F, t, r) with confidence intervals, effect sizes, degrees of freedom and P value noted<br>*Give P values as exact values whenever suitable.*
☒	☐	For Bayesian analysis, information on the choice of priors and Markov chain Monte Carlo settings
☒	☐	For hierarchical and complex designs, identification of the appropriate level for tests and full reporting of outcomes
☐	☒	Estimates of effect sizes (e.g. Cohen's d, Pearson's r), indicating how they were calculated

Our web collection on statistics for biologists contains articles on many of the points above.

Software and code

Policy information about availability of computer code

Data collection	Optical density measurements in a BioTek Epoch 2 microplate reader were collected with the manufacturer software Gen5 3.09.<br>Model simulations were conducted in Python 3.8. The code is available at https://github.com/nnordhol/ODEvolution .
Data analysis	Variant calling was done using the breseq-0.33 pipeline. Downstream analysis were done in Python 3.8. Growth rates from optical density measurements were calculated in Python 3.6, using a method published by Swain et al (see References in the main text). Statistical analyses were conducted in Python 3.8, using the scipy.stats 1.5.0 module. Data visualisation was done with the seaborn 0.10.1 package in Python 3.8. Images of motility assays were analysed with FIJI 1.53c.

For manuscripts utilizing custom algorithms or software that are central to the research but not yet described in published literature, software must be made available to editors and reviewers. We strongly encourage code deposition in a community repository (e.g. GitHub). See the Nature Research guidelines for submitting code & software for further information.

Data

Policy information about availability of data

All manuscripts must include a data availability statement. This statement should provide the following information, where applicable:

- Accession codes, unique identifiers, or web links for publicly available datasets
- A list of figures that have associated raw data
- A description of any restrictions on data availability

Source data for figures 1, 2, 4, and 5 are provided with this paper. The whole genome sequencing data generated in this study have been deposited in the NCBI database under project ID PRJNA735069. The reference sequence of E. coli K12 MG1655 was obtained from the NCBI RefSeq database (RefSeq accession: NC_000913.3; RefSeq assembly accession: GCF_000005845.2).

Field-specific reporting

Please select the one below that is the best fit for your research. If you are not sure, read the appropriate sections before making your selection.

☒ Life sciences ☐ Behavioural & social sciences ☐ Ecological, evolutionary & environmental sciences

For a reference copy of the document with all sections, see nature.com/documents/nr-reporting-summary-flat.pdf

Life sciences study design

All studies must disclose on these points even when the disclosure is negative.

Sample size	When statistical analyses were conducted, experiments were done at least in triplicate and sample sizes were chosen to resolve meaningful differences between groups based on the expected variation of a particular experimental method observed in preliminary experiments in our lab (survival fractions, growth rates, cytochrome c assay) or in the literature (e.g. biofilm assay, PMID: 21307833). Exact sample sizes are reported in the figure legends. The chosen sample sizes are similar to those commonly used in the field of persistence and experimental evolution (PMID: 27572640, PMID: 25043002, PMID: 28183996, PMID: 30287875).
Data exclusions	Samples in which no colony forming units could be detected in the time kill assays in Figure 1 and Figure 5 were excluded from statistical analysis but indicated on the x-axis. The rationale was that it was not possible to determine wheter these samples did not contain any cells ("true zeros") or whether the cell concentration was below the detection limit.
Replication	All experiments were replicated with multiple independent biological samples. The number of independent biological samples is indicated in the figure legends.
Randomization	Randomization was not possible because the samples were handled by an individual experimenter.
Blinding	Blinding was not applicable because experimental groups did not consist of humans and samples were handled by an individual experimenter.

Reporting for specific materials, systems and methods

We require information from authors about some types of materials, experimental systems and methods used in many studies. Here, indicate whether each material, system or method listed is relevant to your study. If you are not sure if a list item applies to your research, read the appropriate section before selecting a response.

Materials & experimental systems

n/a	Involved in the study	
☒	☐	Antibodies
☒	☐	Eukaryotic cell lines
☒	☐	Palaeontology and archaeology
☒	☐	Animals and other organisms
☒	☐	Human research participants
☒	☐	Clinical data
☒	☐	Dual use research of concern

Methods

n/a	Involved in the study	
☒	☐	ChIP-seq
☒	☐	Flow cytometry
☒	☐	MRI-based neuroimaging
